# Upregulation of miR-23a∼27a∼24-2 Cluster Induces Caspase-Dependent and -Independent Apoptosis in Human Embryonic Kidney Cells

**DOI:** 10.1371/journal.pone.0005848

**Published:** 2009-06-09

**Authors:** Ravindresh Chhabra, Yogita K. Adlakha, Manoj Hariharan, Vinod Scaria, Neeru Saini

**Affiliations:** Institute of Genomics and Integrative Biology, Delhi, India; Roswell Park Cancer Institute, United States of America

## Abstract

miRNAs have emerged as important players in the regulation of gene expression and their deregulation is a common feature in a variety of diseases, especially cancer. Currently, many efforts are focused on studying miRNA expression patterns, as well as miRNA target validation. Here, we show that the over expression of miR-23a∼27a∼24-2 cluster in HEK293T cells induces apoptosis by caspase-dependent as well as caspase-independent pathway as proved by the annexin assay, caspase activation, release of cytochrome-c and AIF (apoptosis inducing factor) from mitochondria. Furthermore, the over expressed cluster modulates the expression of a number of genes involved in apoptosis including FADD (Fas Associated protein with Death Domain). Bioinformatically, FADD is predicted to be the target of hsa-miR-27a and interestingly, FADD protein was found to be up regulated consistent with very less expression of hsa-miR-27a in HEK293T cells. This effect was direct, as hsa-miR-27a negatively regulated the expression of FADD 3′UTR based reporter construct. Moreover, we also showed that over expression of miR-23a∼27a∼24-2 sensitized HEK293T cells to TNF-α cytotoxicity. Taken together, our study demonstrates that enhanced TNF-α induced apoptosis in HEK293T cells by over expression of miR-23a∼27a∼24-2 cluster provides new insights in the development of novel therapeutics for cancer.

## Introduction

miRNAs are a class of highly conserved, non-coding single-stranded RNAs (18–24 nt) derived from the endogenously produced pre-miRNA (precursor) having a hairpin (stem-loop) structure [1]–[3]. miRNAs refine gene expression post-transcriptionally either via the cleavage of target mRNAs or the inhibition of translation of target mRNAs depending upon complementarity between miRNA and the 3′ untranslated regions (UTRs) of targeted mRNA in animals and plants [4]–[8]. The emerging role of miRNAs in the regulation of fundamental set of cellular mechanisms such as proliferation, apoptosis, development, differentiation and metabolism [9]–[16] clearly suggests that any aberration in miRNA biogenesis pathway or its activity contributes to the human disease pathogenesis including cancer [17]. Recent findings have also demonstrated that miRNAs could act either as potential oncogenes or tumor suppressor genes [18], [19].

Apoptosis, or programmed cell death, is a highly regulated, morphologically and biochemically distinct physiological cell death event which is accompanied by cytoskeletal disruption, cell shrinkage, membrane blebbing, chromatin condensation, and internucleosomal DNA fragmentation [20]. Caspases, a family of aspartate-specific cysteine proteases, play a critical role in the execution of apoptosis. Broadly caspases can be divided into two types - Initiator and Effector. Initiator caspases, with long prodomains, such as caspases-8, -9 and -10, either directly or indirectly activate effector caspases, such as caspases-3, -6 and -7. These effector caspases then cleave intracellular substrates, including structural and regulatory proteins such as cytokeratin, poly (ADP-ribose) polymerase (PARP) and are directly responsible for many of the morphological features of apoptosis [21]. Literature also shows that cells can undergo apoptosis in caspase-dependent and/or -independent manner. Caspase-dependent pathway can be further divided based on the initiator caspases into the extrinsic pathway or intrinsic pathway. The extrinsic pathway (the death receptor-mediated pathway) is triggered by the members of the tumor necrosis factor (TNF) receptor super family. Binding of the ligand to its cognate receptor results in receptor trimerisation and subsequent recruitment of the dual adaptor molecule FADD leading to rapid activation of caspase-8 through recruitment of procaspase-8. Intrinsic pathway (the mitochondrial-mediated pathway) is activated in response to a variety of stress conditions, such as cytotoxic drugs, UV irradiation or growth factor withdrawal, which induce mitochondrial perturbation and the cytoplasmic release of pro-apoptotic mitochondrial proteins such as cytochrome-c leading to the activation of caspase-9. Both pathways finally lead to activation of caspase-3, the executioner caspase responsible for the final morphological changes observed during apoptosis. Accumulating evidences suggest that a cross-talk between two pathways exists in cells as the active caspase-8 cleaves a BH3-domain-only subfamily protein, Bid. This truncated Bid (tBid) translocates to mitochondria and mediates cytochrome-c release [22]. Caspase- independent pathway occurs via the translocation of AIF (Apoptosis Inducing Factor) from the mitochondria towards the nucleus; ultimately resulting in apoptosis [23].

Resistance towards apoptosis is a key factor for the survival of a malignant cell. Cancer results if there is too little apoptosis and cells grow faster and live longer than normal cells. In addition, defects in apoptosis signaling contribute to drug resistance of tumor cells. Thus, one of the main goals for oncologic treatment is to overcome resistance of tumor cells towards apoptosis. The exciting challenge in oncology is to translate the growing knowledge of apoptotic pathways into clinical applications.

The objective of the present study was to examine the role of the miRNA cluster (miR-23a∼27a∼24-2) in apoptosis. Understanding the role of miRNA in apoptosis is likely to provide deeper insights into various disease processes and thus totally revolutionize the scientific approach towards developing therapies against these diseases. Since their discovery, a number of bioinformatic softwares have come up predicting the targets of miRNAs, yet very few miRNA-target interactions have been validated experimentally.

Here, we show that the over expression of miR-23a∼27a∼24-2 cluster in HEK293T cells induces apoptosis by caspase-dependent as well as -independent pathway. We also show that hsa-miR-27a negatively regulated the expression of FADD.

FADD/Mort1 is a cytosolic adaptor molecule which is critical for signaling from CD95 (FAS/Apo1) and certain other members of the tumor necrosis factor receptor (TNF-R) family (called death receptors). Two protein interaction domains have been identified in FADD. The C-terminal “death domain” (DD) is needed for recruitment of FADD/Mort1 to ligated death receptor and N-terminal “death effector domain” (DED) mediates oligomerization and activation of caspase-8 zymogens. Caspase-8 activates other caspases by cleavage and this starts a proteolytic cascade which constitutes the “point of no return” in apoptotic signaling. Besides its role in apoptosis, this protein has also been implicated in survival/proliferation and cell cycle progression. FADD can also function as tumor suppressor gene [25]. Many reports also reveal the underexpression of FADD in several cancers like thyroid adenoma and acute myeloid leukemia [26], [27]. Lack of FADD protein confers survival/growth advantages in tumor cells.

The diverse roles of FADD ranging from cell survival to cell death and little knowledge of regulation of FADD expression makes it an interesting subject to study and we believe that finding a miRNA targeting this gene would add a new dimension to the ongoing research in the cancer therapeutics.

## Materials and Methods

### Sequences retrieval, Primer Designing and Cloning

The sequences of miRNAs were retrieved from miRbase *(* http://microrna.sanger.ac.uk/sequences/ *)*, the 3′ UTR sequence was retrieved from NCBI *(* http://www.ncbi.nlm.nih.gov/sites/entrez/ *)* and for primer designing primer 3 software was used *(* http://frodo.wi.mit.edu/ *)*. We cloned 3′ UTR of FADD gene into pMIR-REPORT miRNA Expression Reporter Vector (Ambion, Austin, TX, USA) between SpeI and HindIII restriction sites using forward primer CCG CCG ACT AGT CAG CCT GGA CTT TGG TTC TC and reverse primer CCG CCG AAG CTT CTG CCT TGG CAA TTC TGT TA. miR-23a∼27a∼24-2 sequence was amplified by PCR from genomic DNA using the following primers: CCG CCG AAG CTT CAT CTC TGC TCC AAG CAT CA and CCG CCG GGA TCC TCT CTT TCT CCC CTC CAG GT and cloned in pSilencer4.1 vector (Ambion, Austin, TX, USA). The resulting plasmids were sequenced to ensure accuracy.

### Cell Culture and Transfection

A549 (NSCLC, lung adenocarcinoma), HepG2 (hepatocellular adenocarcinoma), HeLa (cervical carcinoma), H520 (lung carcinoma), HEK293T (human embryonic kidney), MCF7 (breast carcinoma), Hep2 (laryngeal carcinoma) and Neuro2A (rat neuronal) cells were obtained from National Centre for Cell Science, Pune, India and maintained in DMEM medium containing 10% (v/v) fetal calf serum, 100 units/ml penicillin, 100 µg/ml streptomycin, 0.25 µg/ml amphotericin in a humidified 5% CO_2_ atmosphere. For luciferase assay, the cells were cotransfected in 6-well plates by using lipofectamine 2000 (Invitrogen, CA, USA) according to the manufacturer's protocol with 1 µg of the luciferase reporter vector, 1 µg of pSilencer4.1 and 0.3 µg of the β-gal vector (for normalization of transfection, Ambion Austin, TX, USA). For each well 40 nM/80 nM anti-miR-27a (Ambion Austin, TX, USA) was used as indicated. Luciferase and β-gal assays were done simultaneously on the cells 24 h after transfection using Promega kits. For over expression studies, the cells were cotransfected in 6-well plates by using lipofectamine 2000 with either 2 µg or 4 µg of the pSilencer 4.1 expressing the miRNA cluster.

### Semiquantitative RT-PCR analysis

Total RNA was extracted using Trizol reagent (Invitrogen, CA, USA). Reverse transcription was carried out with M-MuLV reverse transcriptase (MBI Fermentas, USA) according to the manufacturer's protocol using 2 µg of total RNA. Primers for miR-23a (CCG CCG GGA TCC ACG GCC GGC TGG GGT TCC and CCG CCG AAG CTT CAG AGC TCA GGG TCG GTT GG), mir-27a (CCG CCG GGA TCC AGC TCT GCC ACC GAG GAT and CCG CCG AAG CTT AGG ATG GCA GGC AGA CAG), miR-24-2 (CCG CCG GGA TCC CTG TCT GCC TGC CAT CCT and CCG CCG AAG CTT CAT CTC TGC TCC AAG CAT CA) and β-actin (TGC GTG ACA TTA AGG AGA AG and CAT TGC CGA CAG GAT GCA G) were designed. The PCR conditions used were as follows: initial denaturation at 94°C for 5 min followed by 30 cycles of 94°C for 30 sec, 56°C for 45 sec, 72°C for 45 sec, and an additional cycle with extension at 72°C for 5 min. The normalization was done using β-actin RT-PCR.

### RT-PCR and real-time TaqMan PCR

Total RNA was extracted using Trizol reagent and reverse transcription was carried out with M-MuLV reverse transcriptase according to the manufacturer's protocol. Primers for pre-miR-24-2 (CTC CCG TGC CTA CTG AGC T and CCC TGT TCC TGC TGA ACT GAG), pre-miR-27a (GCA GGG CTT AGC TGC TTG and GGC GGA ACT TAG CCA CTG T) and 18 s (GTA ACC CGT TGA ACC CCA TT and CCA TCC AAT CGG TAG TAG CG) were designed. Analyses were carried out using SYBR Green PCR master mix (Applied Biosystems, Foster City, CA). Results were normalized with 18 s rRNA.

TaqMan microRNA assays (Applied Biosystems, Foster City, CA) that include specific RT primers and TaqMan probes were used to quantify the expression of mature miR-27a (PN4373287) and miR-24-2 (PN4373072). 18 s rRNA (PN 4333760F) was used for normalization. The real time PCR data was normalized using Pfaffl's method [28].

### Annexin-V assay

Surface exposure of phosphatidylserine in apoptotic cells was measured by Guava Nexin Kit according to the manufacturer's protocol (Guava Technologies, Hayward, California, USA). Annexin-PE fluorescence was analyzed by cytosoft software (Guava Technologies, Hayward, California, USA). Wherever indicated HEK293T cells were pre-treated with BOC-D-FMK (broad caspase inhibitor, 300 µM, Sigma, St. Louis, Missouri, USA) for 30 min before transfection.

### Measurement of intracellular ROS generation, mitochondrial membrane potential (Δψm) and cytochrome-c release

Mitochondrial membrane potential was measured using DiOC6 (3,3′-dihexyloxacarbocyanine iodide) dye and ROS production was measured using DCFH-DA (2′,7′-dichlorfluorescein-diacetate) by flow cytometry as described before [29]. In brief, untransfected and p(23a∼27a∼24-2) transfected HEK293T cells were washed with PBS and DCFH-DA (5 µM) or fluorochrome DiOC6 (40 nM) was added 30 min prior to harvesting. Fluorescence intensity was monitored using flow cytometer (Guava Technologies, Hayward, California, USA). A minimum of 10,000 events were counted.

Cytochrome-c release was observed using Cell Fractionation Kit from BD Biosciences [30]. In brief, untransfected and p(23a∼27a∼24-2) transfected HEK293T cells were trypsinized, harvested, washed once with PBS and resuspended in 100 µl of lysis buffer. The cells were then homogenized and centrifuged to separate mitochondrial pellet and cytosolic fraction. Cytochrome-c release was analyzed in cytosolic and mitochondrial fraction by immunoblotting. Cox IV was used to check the purity of cytosolic and mitochondrial fraction. Equal loading was confirmed using β-actin.

### Protein preparation and western blot analysis

Untransfected and p(23a∼27a∼24-2) transfected HEK293T cells were trypsinized and cell pellets were lysed with modified RIPA buffer (50 mM Tris-HCl, pH 7.4, 150 mM NaCl, 1 mM EDTA, 1% NP40, 0.25% Na deoxycholate, 1 µg/ml aprotinin, 1 µg/ml leupeptin, 1 µg/ml pepstatin, 1 mM PMSF, 1 mM sodium orthovanadate, and 1 mM sodium fluoride) and kept in ice for 30 min. Lysate was centrifuged at 12000 rpm for 30 min and supernatant was collected. Protein concentration was determined using the Bio-Rad protein assay kit. Equal amounts of sample lysates (40 µg) were separated by 12% sodium dodecyl sulphate - polyacrylamide gel electrophoresis (SDS-PAGE) and transferred to PVDF membrane (mdi, Advanced Microdevices, India). The membrane was blocked with 3% skim milk in Tris buffered saline (20 mM Tris, 150 mM NaCl, pH 7.4) with 0.1% Tween-20 for 1 h and then incubated with primary antibody (FADD/caspase 8/caspase 3/PARP/Bid/Bcl-2/Bax) in 1% skim milk for 2 h followed by incubation with appropriate secondary antibody (anti-mouse ALP linked or/anti-rabbit HRP linked) for 1 h. Blots were developed using enzyme based chemiluminescence assays (Alkaline phosphatase or Horse-radish peroxidase) using NBT- BCIP or DAB as substrate. Equal loading of protein was confirmed using β-actin antibody or GAPDH antibody. All experiments were repeated at least three times; representative results are presented [31].

### Quantitiative and Statistical analysis of western blot data

Measurement of signal intensity on PVDF membranes after western blotting with various antibodies was performed using AlphaImager 3400 (Alpha InnoTech Corporation, San Leandro, California). All data were expressed as integrated density values (IDV). For FADD, Caspase 3, Caspase 8, Bcl-2, Bax, Bid, PARP the IDV values were calculated as the density values of the specific protein bands/β actin or GAPDH density values. All figures showing quantitative analysis include data from atleast three independent experiments. Statistical analysis was performed with student's two tailed t-test using SPSS (windows version 7.5); values of p≤0.05 were considered statistically significant.

### Northern Blotting

Total RNA was extracted with the Trizol reagent as per the manufacturer's instructions. For Northern blotting, the total RNA (40 µg) was run on a 15% polyacrylamide-urea gel, transferred to a Hybond N^+^ membrane using semidry apparatus (BioRad, Hercules, CA, USA) and UV-crosslinked (Stratalinker). The blot was probed with a RNA probe using miRVana probe construction kit (Ambion Austin, TX, USA). The blot was scanned in the phosphorimager after overnight exposure. The normalization of the result was done by stripping the blot in 0.01% SDS at 80°C for 2 hours and probing it for U6 expression.

### Probe sequence for 27a and U6

Probe 27a: TTC ACA GTG GCT AAG TTC CGC CCT GTC TC

Probe U6: TGC TAA TCT TCT CTG TAT CG

### Luciferase assay

Twenty-four hours before transfection, HEK293T cells were plated in 6-well plate. pMIR-REPORT miRNA Expression Reporter Vector containing 3′UTR region of the FADD and/or p-Silencer 4.1 vector expressing hsa-miR-23a∼27a∼24-2 (p(23a∼27a∼24-2)) were transfected into HEK293T cells using lipofectamine 2000 (Invitrogen, CA, USA) as described by the manufacturer. pCDNA 3.1/anti-miR-23a/anti-miR-27a/anti-miR-24-2 (40 nm/80 nm Ambion, Austin, TX, USA) were also used wherever indicated. Luciferase activity was measured using Luciferase Kit Assay System (Promega, Madison, WI, USA) and readings were taken on the luminometer (Berthold AutoLumat LB953 rack luminometer). Luciferase activity was then normalized by β-galactosidase activity for transfection in each well.

### Reporter assay

The pNF-κB-luc reporter plasmid containing direct repeats of the transcription recognition sequence for NF- κB (path detect NF- κB Cis-Reporting system) was a kind gift from Zdenek Dvorak. For reporter assay, untransfected and p(23a∼27a∼24-2) transfected HEK293T cells were taken and following 8 h incubation period, cells were treated with TNF-α (10 ng/ml) for 16 h. At the end of treatment, cells were lysed and luciferase activity was measured and standardized per milligram of protein [32].

### Immunostaining and confocal microscopy

Cells were seeded on coverslips in six-well plate (Tarsons) at 1.6×10^5^ to 2×10^5^ per well for 24 h prior to transfection. Transfection was done with p(23a∼27a∼24-2) using lipofectamine 2000 as described by the manufacturer. Cells were rinsed with PBS and fixed with ice-cold acetone: methanol mix (1∶1) for 10 min at −20°C. They were rinsed again and permeabilized with 0.5% Triton X-100 in PBS for 15 min at room temperature. This was followed by washing with PBS and consequently blocking for 45 min in 1% bovine serum albumin in PBS at room temperature, washed with PBS, and incubated with anti-AIF antibody (1∶50, Santa Cruz Biotechnology) for 1 h 30 min at room temperature. Then, cells were washed with PBS and incubated with the secondary antibody anti-mouse-TRITC conjugated (1∶200, Bangalore Genei) for 45 min at room temperature. Finally, cells were washed with PBS and stained with DAPI (0.5 ug/ml) for 10 min. Fluorescent images were acquired through a Confocal microscope (Carl Zeiss, Germany) [33].

## Results

### Expression of miR-23a, miR-27a and miR-24-2 in HEK293T cells after p(23a∼27a∼24-2) cluster over expression

**Figure 1:**
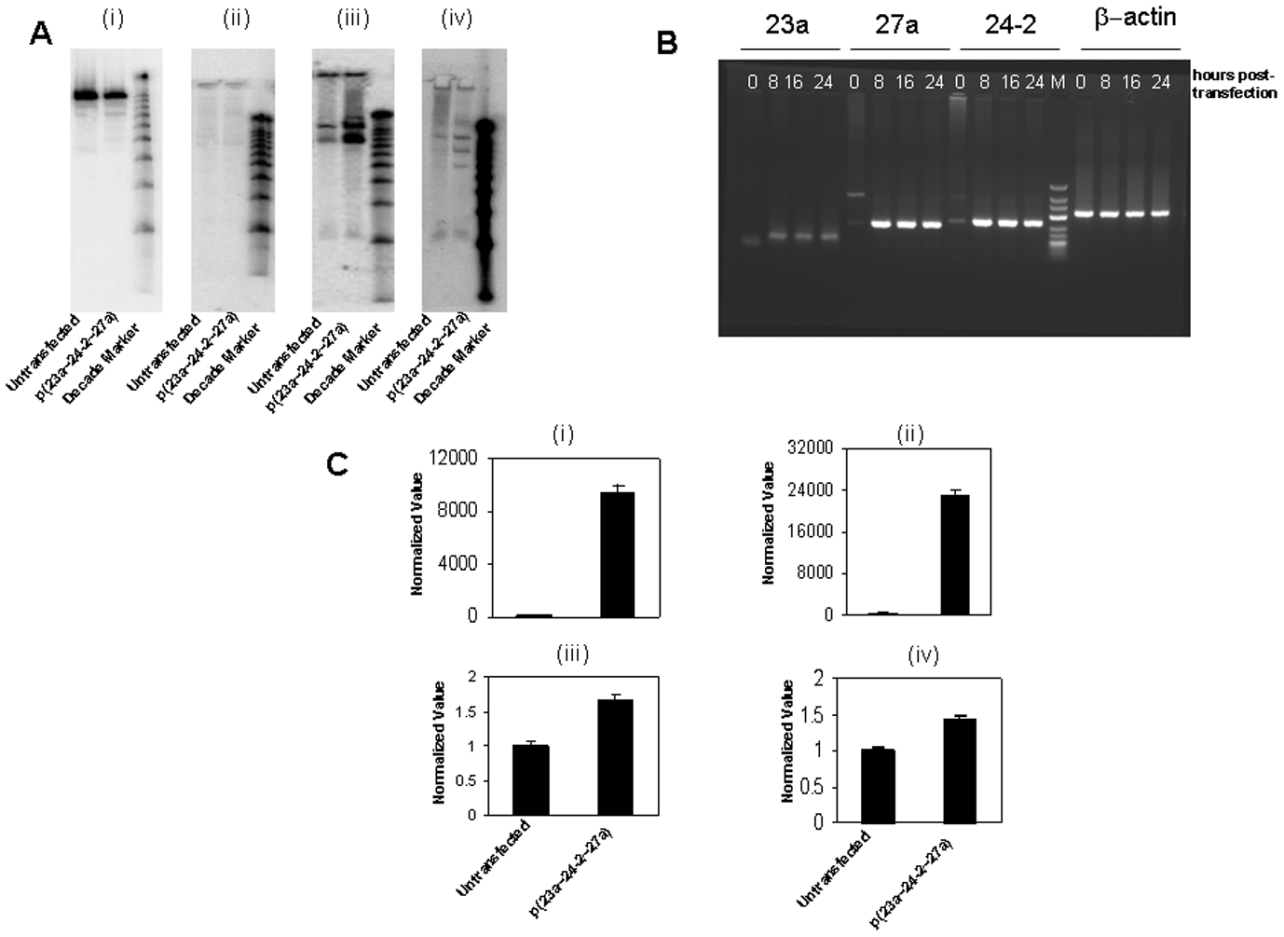
Overexpression of p(23a∼27a∼24-2) in HEK293T cells causes a change in precursor and mature form of mir-23a, miR-27a and miR-24-2. A. Northern Blot depicting change in miRNA expression after p(23a∼27a∼24-2) transfection (i) U6 loading control (ii) miR-23a (iii) miR-27a (iv) miR-24-2. B. Semiquantitative PCR confirms the increase in the miRNA transcript levels of miR-24-2 and miR-27a but not much change in miR-23a after p(23a∼27a∼24-2) transfection at 8-, 16- and 24- hours post-transfection, M refers to DNA ladder. The constitutive expression of β-actin is shown as the loading control. C. Real Time PCR for (i) pre-miR-24-2, (ii) pre-miR-27a and TaqMan assay for (iii) Mature miR-24-2, (iv) Mature miR-27a.

We first did northern blot analysis to determine the expression of miR-23a, miR-27a and miR-24-2 in HEK293T cells. As shown in Fig. 1A, northern blot analysis for microRNAs showed that only microRNA 27a was abundantly expressed in the mature form in HEK293T cells after over expression of p(23a∼27a∼24-2) cluster. MicroRNA 23a levels were not detectable and there was no change in the mature form of microRNA 24-2 before and after p(23a∼27a∼24-2) cluster over expression.

To better address the expression levels of miR-23a, miR-27a and miR-24-2 in HEK293T cells before and after p(23a∼27a∼24-2) cluster over expression, we did time course study using semiquantitative PCR. Fig. 1B shows that expression of microRNA 23a was very low whereas the expression levels of microRNA 24-2 and microRNA 27a were significantly high after p(23a∼27a∼24-2) cluster over expression in HEK293T cells as compared to untransfected HEK293T cells. To further confirm the semiquantitative PCR result, we did SYBR green based real time PCR assay for the precursor form and TaqMan based real time PCR assay for the mature form of miR-24-2 and miR-27a. As inferred from the Fig. 1C (i, ii), there was a very significant increase in the precursor form of both miR-24-2 and miR-27a and there was 1.66 fold increase in the levels of the mature form of microRNA 24-2 and 1.42 fold increase in the levels of the mature form of microRNA 27a (Fig. 1C iii, iv) after p(23a∼27a∼24-2) cluster over expression in HEK293T cells as compared to untransfected HEK293T cells.

### p(23a∼27a∼24-2) induces apoptosis in HEK293T cells

**Figure 2:**
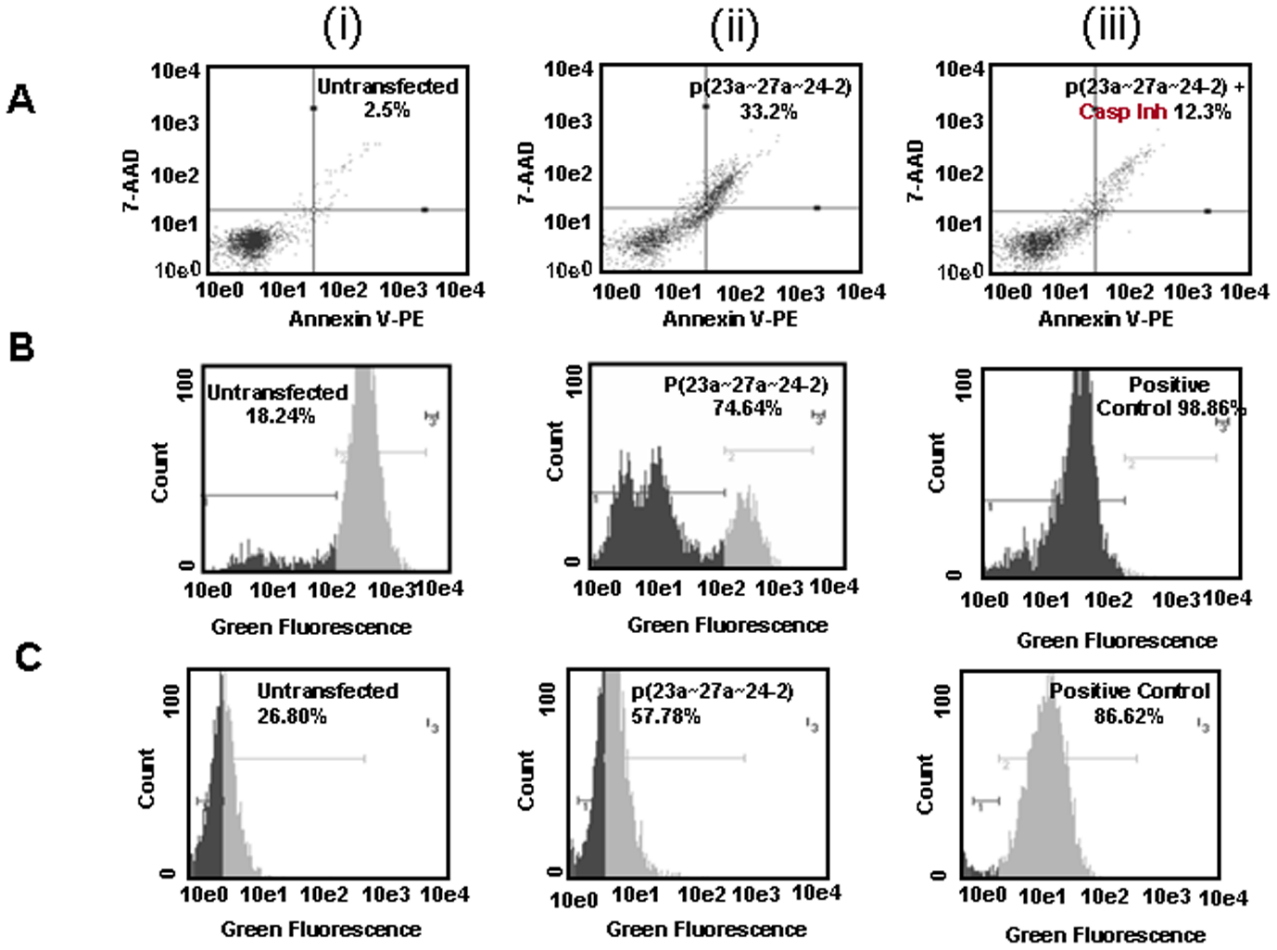
Biological effect of over expression of p(23a∼27a∼24-2) in HEK293T cells. A. Annexin V-PE binding in HEK293T cells i) untransfected cells, ii) cells transfected with 4 µg p(23a∼27a∼24-2) for 24 h, and iii) cells transfected with p(23a∼27a∼24-2) for 24 h and treated with caspase inhibitor. B. Mitochondrial membrane potential in HEK293T cells, i) untransfected cells, ii) 24 h after transfection with 4 µg p(23a∼27a∼24-2), and iii) positive control, detected by DiOC6 staining and flow-cytometry analysis. C. ROS production in HEK293T cells i) untransfected cells, ii) 24 h after transfection with 4 µg p(23a∼27a∼24-2), and iii) positive control.

A number of studies have demonstrated that microRNAs can act as oncogenes or tumor suppressors [34]–[35]. Infact, the same microRNA can act as an oncogene in one biological condition and as a tumor suppressor gene in another [18], [36]. Recent report suggests that upregulation of miR-23a∼27a∼24-2 cluster decreases transforming growth factor-beta induced tumor suppressive activities in human hepatocellular carcinoma cells [37]. In the present study we wanted to investigate the biological effects of over expression of miR-23a∼27a∼24-2 cluster in HEK293T cells. As shown in Fig. 2A, the annexin positive cells increased to 33.2%±0.8% (p = 0.024) after over expression of miRNA cluster in HEK293T as compared to 2.5%±0.3% (p = 0.016) in untransfected HEK293T cells. Moreover, in the presence of caspase inhibitor (BOC-D-FMK) the annexin positive cells came down to 12.3%±0.4% (p = 0.015). The results of annexin assay were also validated with a positive control (data not shown). Our findings show that the over expression of this miR-23a∼27a∼24-2 cluster induces apoptosis in HEK293T cells by caspase-dependent as well as caspase-independent pathway.

Since mitochondria plays a major role in both the types of apoptosis, we examined disruption in the mitochondrial membrane potential (Δψm) by DiOC6 and ROS production by DCFH-DA in untransfected and p(23a∼27a∼24-2) transfected HEK293T cells. As indicated in Fig. 2B in comparison to controls where only 18.24% (p<0.002) of cells shifted towards left, approximately 74.64% cells (p<0.04) shifted towards left in miR-23a∼27a∼24-2 transfected HEK293T cells. Our results indicate that over expression of miR-23a∼27a∼24-2 cluster induces significant disruption of Δψm in HEK293T cells. mCICCP was used as positive control for DiOC6 experiments.

The DCFH-DA assay (Fig. 2C) also shows that over expression of miR-23a∼27a∼24-2 cluster increases ROS production as we observed an increase in fluorescence intensity from 26.80% (p<0.03) in untransfected HEK293T cells to 57.78% (p<0.02) in miR-23a∼27a∼24-2 cluster over expressed HEK293T cells.

### p(23a∼27a∼24-2) modulates expression of proteins of apoptotic pathways

**Figure 3:**
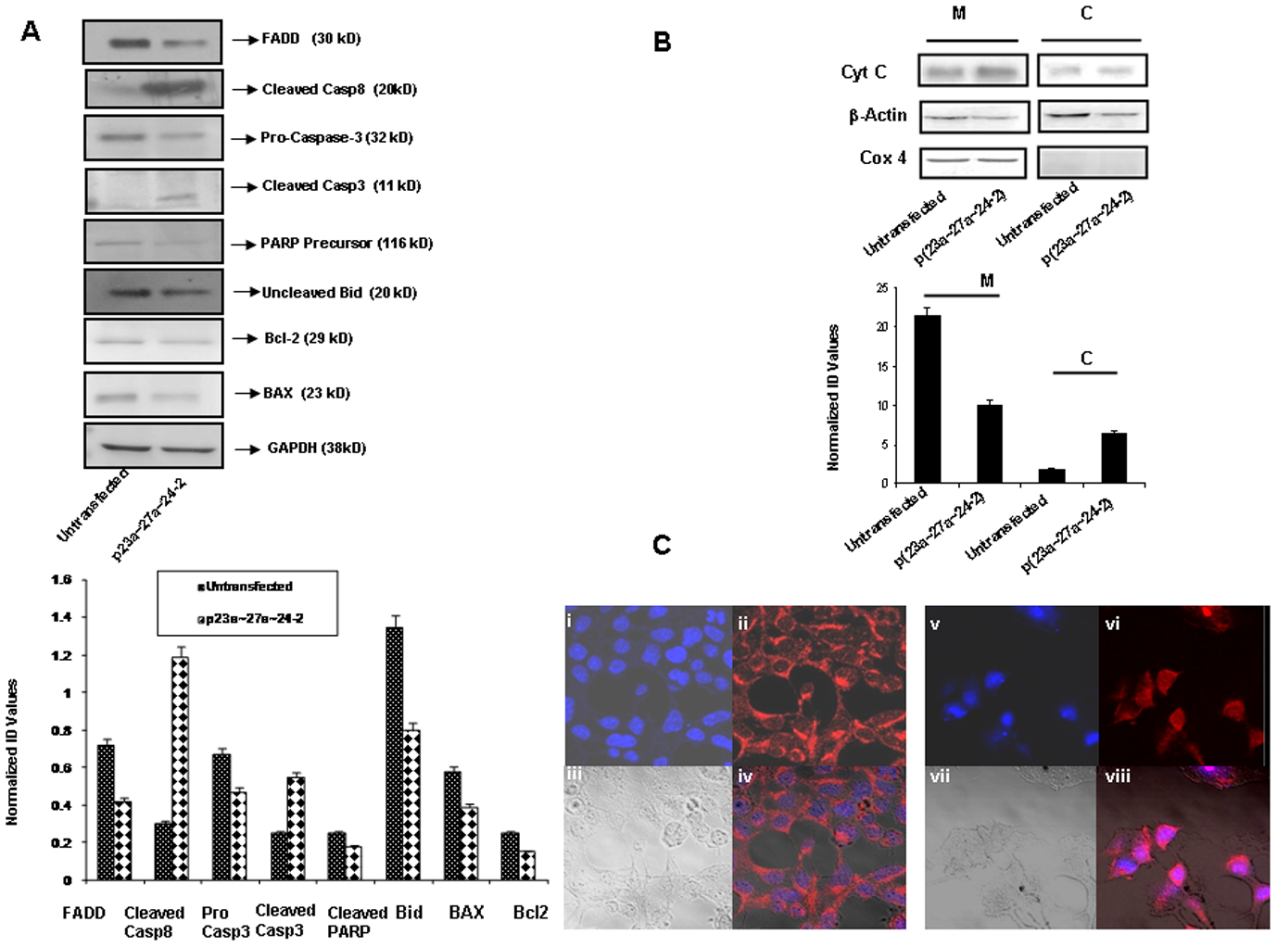
p(23a∼27a∼24-2) modulates pro and anti-apoptotic proteins in HEK293T cells. A. Effect of overexpressed miRNA cluster on the protein expression of FADD, Caspase 8, Caspase 3, Bcl-2, PARP, Bax and Bid in HEK293T cells. GAPDH/β-actin was used as an internal control. Data are representative of a typical experiment repeated three times with similar results. B. Release of Cytochrome-c from mitochondria to cytoplasm after the over expression of miR-23a∼27a∼24-2. Mitochondrial and cytoplasmic fractions were separated as described in the method section. The purity of the fraction was determined by the expression of Cox 4 (mitochondrial specific protein). C. Immunocytochemistry of AIF (red) after over expression of miRNA cluster in HEK293T. Nuclei were counterstained with the DNA-binding dye DAPI (4′,6-diamidino-2-phenylindole; blue). Merged images are shown in iv and viii, bright field images are shown in iii and vii.

In order to better understand the molecular basis of this cluster induced apoptosis, we checked the expression of various proteins involved in apoptosis before and after the over expression of this cluster in HEK293T cells. Our western blot analysis (Fig. 3A) showed 0.70 fold (p = 0.02) decrease in procaspase-3 level, 2.20 fold (p = 0.01) increase in cleaved caspase-3 level, 0.72 fold (p<0.04) decrease in PARP protein (116 kD fragment), 0.60 fold (p = 0.023) decrease in Bcl-2 level, 0.67 fold (p = 0.011) decrease in Bax level, 3.98 fold (p = 0.014) increase in cleaved caspase-8 level, 0.59 fold decrease (p = 0.04) in uncleaved Bid level and 0.50 fold decrease in FADD protein level (p = 0.02).

To further confirm that miR-23a∼27a∼24-2 cluster induces apoptosis by caspase-dependent pathway we did western blot analysis for cytochrome-c. As shown in Fig. 3B, the cytochrome-c levels in the cytosol increased by 3.42 fold (p = 0.03) after the over expression of this cluster.

It is known that apoptosis-inducing factor (AIF), perhaps the best-studied example of a caspase- independent cell death (CICD) mediator, is slowly released from mitochondria following mitochondrial outer membrane permeabilization in a caspase-independent manner although caspase activity may accelerate the release [38]. Following release, AIF translocates to the nucleus. To confirm that the over expression of this cluster induces apoptosis by caspase- independent pathway also, we next performed immunostaining to look for nuclear translocation of AIF (Apoptosis Inducing Factor) and confocal imaging was used to determine the location of AIF in HEK293T cells before and after cluster over expression. We observed that AIF translocates from mitochondria to the nucleus after the cluster over expression (Fig. 3C).

The results obtained here suggest that over expression of this cluster induces FADD independent caspase-8 activation as there was decrease of FADD in miR-23a∼27a∼24-2 transfected HEK293T cells as compared to untransfected cells. Hence, it was confirmed that over expression of this cluster induces apoptosis by both caspase-dependent and -independent pathways.

### FADD is a potential target of hsa-mir-27a

**Figure 4:**
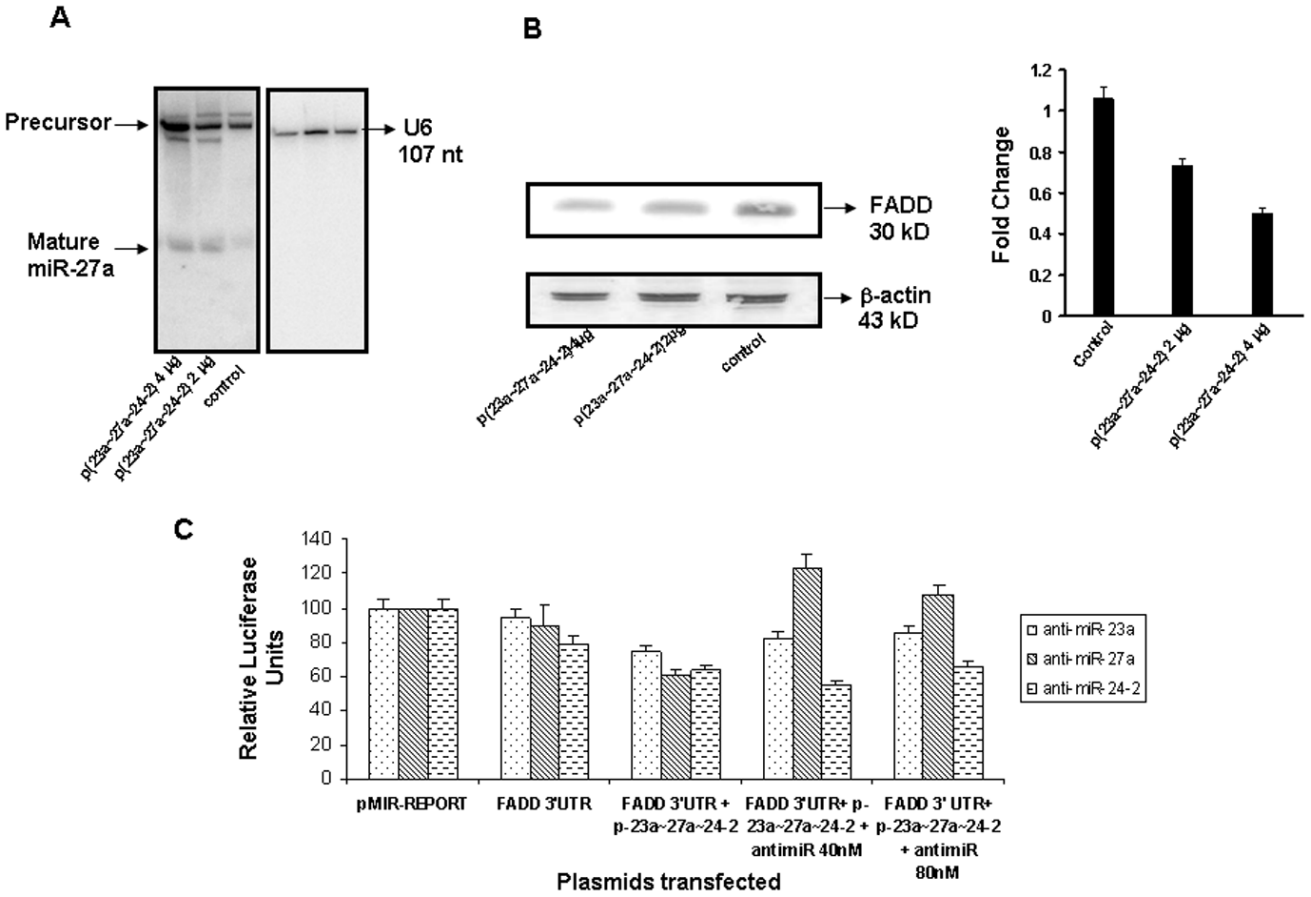
Dose dependent expression of miR-27a and FADD and luciferase assay confirms that miR-27a targets the FADD 3′ UTR. A. Detection of mature miRNA after transfection of clone expressing p(23a∼27a∼24-2), the same blot was probed for U6 expression for normalization. B. Western blotting for FADD after transfection of clone expressing miR-27a, the same blot was probed for β-actin for normalization. The fold change in expression of FADD is also presented after densitometric analysis. C. Luciferase assay proves that 3′UTR of FADD is a target of miR-27a. *p(23a∼27a∼24-2) (2 µg) – Transfection of 2 µg of p(23a∼27a∼24-2). p(23a∼27a∼24-2) (4 µg) – Transfection of 4 µg of p(23a∼27a∼24-2).

MicroRNAs regulate gene expression through decreased translation, increased degradation of the target message, or both [39]. Prediction of animal miRNA targets is challenging because there is only a partial pairing of miRNAs with their targets, and the mechanism of miRNA-mediated regulation is poorly understood. To improve the accuracy of binding site prediction, we used three target prediction algorithms, miRanda [40], RNAhybrid [41] and Target Scan [42]. Only those miR-target pairs predicted by all the three softwares were used in the analysis. The transcript (ENST00000301838) arising from the FADD gene was identified as a target to hsa-miR-27a. We observed that there is perfect complementarity between miR-27a and the 3′UTR of FADD over the seed region (2–9 bases of the mature miRNA). To ascertain if miR-27a regulates FADD, we transfected HEK293T cells with p(23a∼27a∼24-2) and did northern blot and western blot analysis. Northern blot analysis confirmed increased expression of miR-27a following transfection (Fig. 4A). Similarly, HEK293T cells transfected with p(23a∼27a∼24-2) showed reduced FADD protein by western blot (Fig. 4B). Since we observed dose-dependent increase of miR-27a by northern blot and dose-dependent decrease of FADD by western blot, FADD appeared to be a potential target of hsa-miR-27a.

To demonstrate that the negative effect of miR-27a on FADD expression was direct, we cloned the entire wild type 3′UTR of the FADD gene into luciferase reporter vector and did luciferase reporter assay. The luciferase activity assay at 24 h post transfection demonstrated that miR-23a∼27a∼24-2 cluster suppressed luciferase reporter activity by 32% (Fig. 4C). To demonstrate that this suppression in luciferase activity was the effect of miR-27a and not the effect of other two miRNAs (miR-23a and miR-24-2), we used anti-miRs at two different concentrations (40 nM and 80 nM). Results in the Fig. 4C show that suppression was relieved by anti-miR-27a but not by anti-miR-23a and anti-miR-24∼2. Thus, our data confirms that miR-27a directly inhibits expression of FADD by binding to its target sequence.

### miRNA cluster augments the effect of TNF-α

As TNF-α is known to induce apoptosis via the extrinsic pathway and the miRNA cluster (hsa-miR-23a∼27a∼24-2) also induced apoptosis by down regulating FADD, we next investigated whether over expression of miR-23a∼27a∼24-2 cluster has any effect on the sensitivity of HEK293T cells towards TNF-α.

**Figure 5:**
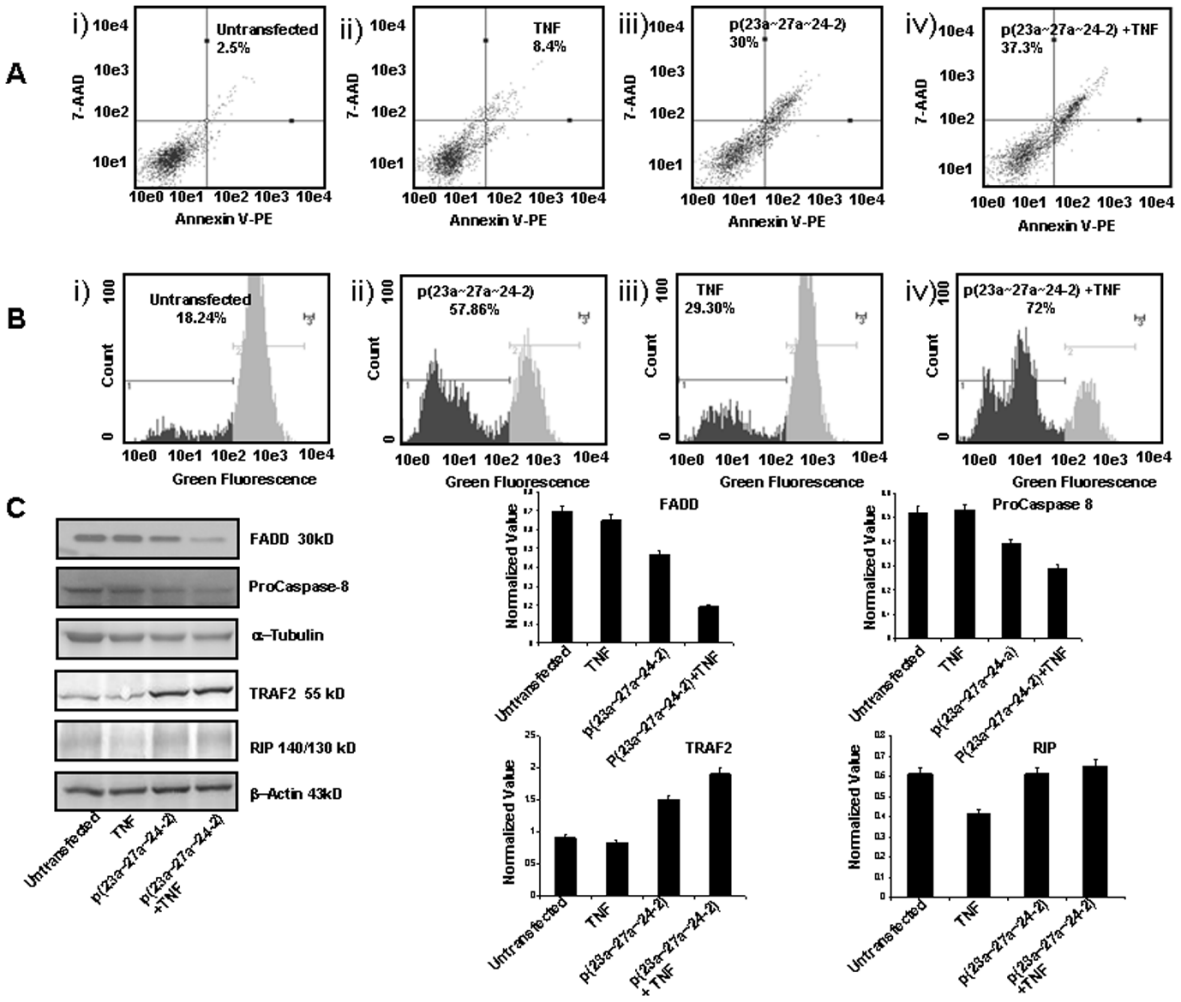
Sensitivity to apoptosis is increased after transfection of p(23a∼27a∼24-2). A. Annexin V-PE binding in HEK293T cells i) untransfected cells, ii) cells transfected with 4 µg p(23a∼27a∼24-2) for 24 h, and iii) cells treated with 20 ng TNF-α iv) cells transfected with 4 µg p(23a∼27a∼24-2) for 24 h and treated with 20 ng TNF-α. B. Mitochondrial membrane potential in HEK293T cells detected by DiOC6 staining and flow-cytometry analysis, i) untransfected cells, ii) 24 h after transfection with 4 µg p(23a∼27a∼24-2), iii) treated with 20 ng TNF-α, and iv) 24 h after transfection with 4 µg p(23a∼27a∼24-2) and treated with 20 ng TNF-α. C. Effect of overexpressed p(23a∼27a∼24-2) on the protein expression of FADD, pro-caspase 8, TRAF2, RIP. GAPDH/β-actin was used as an internal control. Data are representative of a typical experiment repeated three times with similar results.

Our annexin assay (Fig. 5A) showed that p(23a∼27a∼24-2) and TNF-α by themselves induced 30%±0.3% (p = 0.04) and 8.4%±0.5% (p = 0.02) apoptosis, as compared to 37.3%±0.2% (p = 0.018) apoptosis when HEK293T cells were over expressed with the cluster and treated with TNF-α (20 ng/ml) for 18 h. We observed only 2.3% apoptosis in untransfected cells. We also studied their combined effect on the mitochondrial membrane potential (ψ_m_). Our DioC6 assay showed (Fig. 5B) that 72% cells moved towards left when TNF-α treatment was given to cells after over expression of the miRNA cluster as compared to 29% cells which shifted towards left after TNF-α treatment in HEK293T cells and 57% cells that moved towards left in HEK293T cells after over expression with the miRNA cluster. Because receptor interacting protein (RIP), TNF receptor (TNFR)-associated factor 2 (TRAF 2) and the Fas-associated death domain protein (FADD) are important effectors of TNFR1 signaling, we examined whether miR-23a∼27a∼24-2 over expression induces apoptosis through TNFR1. Results in the Fig. 5C show that apart from reducing FADD protein, over expression of miR-23a∼27a∼24-2 cluster in HEK293T cells was causing significant increase in TRAF2 protein levels both in presence or absence of TNF-α. Our western blot data also showed non significant changes in RIP protein levels both in presence or absence of TNF-α.

**Figure 6:**
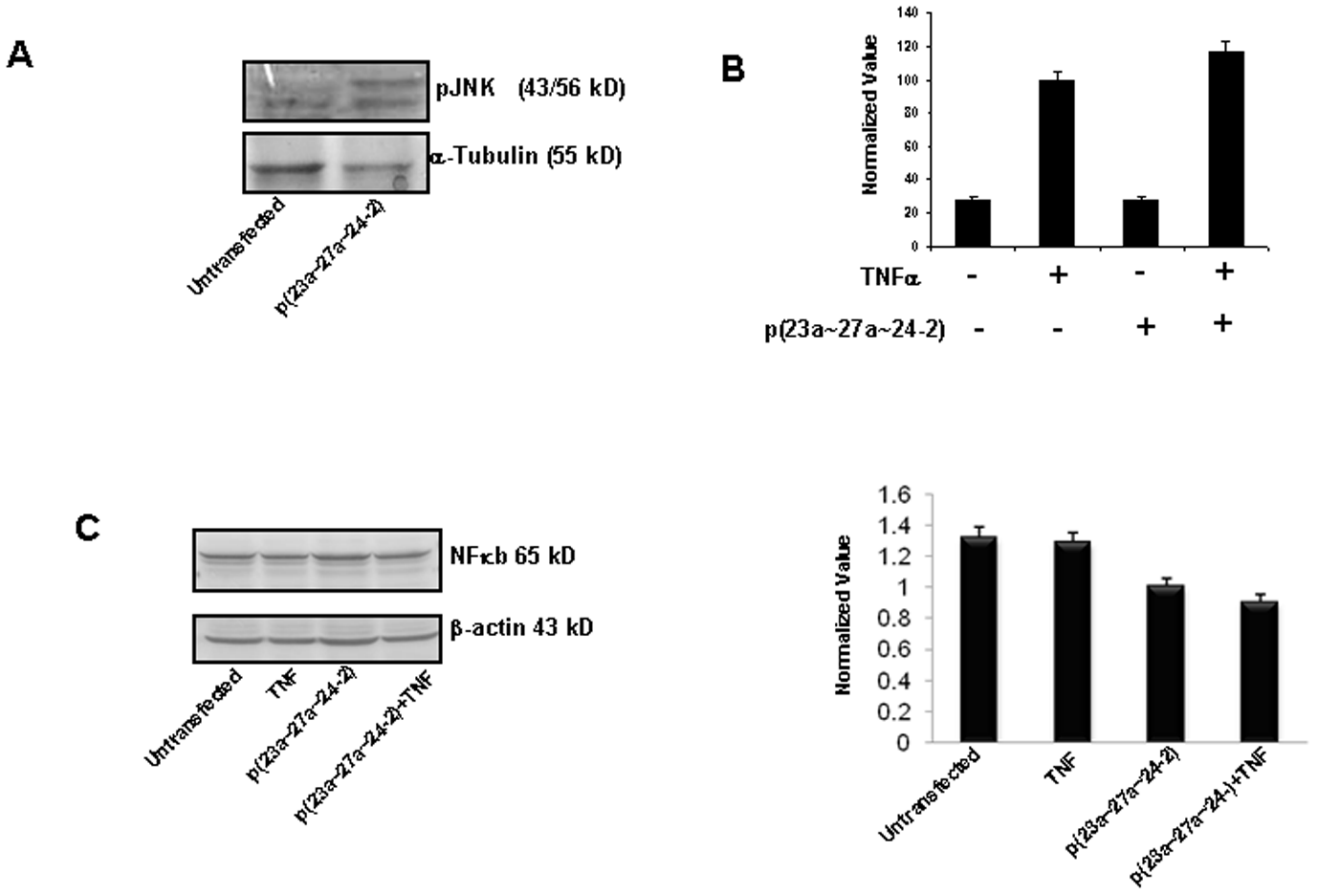
Effect of p(23a∼27a∼24-2) on JNK and NF-κB. A. Western blot analysis for p-JNK before and after p(23a∼27a∼24-2) over expression in HEK293T cells. 50 µg protein was loaded on the gel and blot was developed using BCIP/NBT. Tubulin was used as a loading control. B. Transcriptional activity of NF-κB in transiently transfected HEK293T cells after p(23a∼27a∼24-2) over expression. HEK293T cells were treated with TNF-α after 8 h of transfection and luciferase activity was measured and normalized per mg of protein. C. Western Blot analysis for NF-kB after overexpression of cluster in presence of TNF in HEK293T cells. The fold change in expression of NF-kB is also presented after densitometric analysis.

In literature, it has been found that in RIP and TRAF-2 deficient cells, RIP is most likely responsible for NF-κB activation, whereas TRAF-2 preferentially activates c-Jun N-terminal kinase (JNK) [43]. Since, in our study we observed increased TRAF2 and no change in RIP expression, we postulate that miR-23a∼27a∼24-2 cluster might be inducing apoptosis in HEK293T cells by activating JNK and inhibiting NF-κB. To prove this, we did western blot analysis for p-JNK and NF-κB. (Fig. 6A, C) and also checked for NF-κB transcriptional activity using a reporter assay with pNF-κB-luc plasmid (Fig. 6B). Interestingly, we observed activation of JNK and inhibition of NF-κB in miR-23a∼27a∼24-2 transfected HEK293T cells as compared to untransfected HEK293T cells. It seems that miR-23a∼27a∼24-2 induces apoptosis in HEK293T cells via JNK activation.

## Discussion

The recent report by Gu et al [37] showed that upregulation of miR-23a∼27a∼24-2 cluster decreases transforming growth factor-beta-induced tumor-suppressive activities in human hepatocellular carcinoma cells. Contrary to their findings is our study where for the first time it is shown that upregulation of miR-23a∼27a∼24-2 cluster induces caspase-dependent and caspase-independent apoptosis in human embryonic kidney cells. Consistent to our findings there are reports in the literature where it has been shown that the same microRNA can act as an oncogene under one set of conditions and as a tumor suppressor in another [18], [36].

In this investigation, several observations suggest that miR-27a regulates FADD expression. For example, over expression of miR-23a∼27a∼24-2 cluster in HEK293T cells induces dose-dependent increase of hsa-miR-27a and dose dependent decrease of FADD as seen by northern and western analysis, respectively. This effect was direct, as p(23a∼27a∼24-2) cluster negatively regulated the expression of FADD 3′ untranslated region (UTR)-based reporter construct.

Our data shows that over expression of this cluster induces down regulation of FADD and up regulation of caspase-8 thereby showing that there is FADD-independent caspase-8 activation in HEK293T cells. In regard to caspase-8 activation there are mixed reports. Caspase-8 activation has been extensively studied in apoptosis mediated by members of the death domain receptor family such as Fas/CD95 and TNFR [44]. Interactions between Fas and FADD via their C-terminal death domains expose the N-terminal death effector domain (DED) of FADD, which can interact with DED domains in the caspase-8 proform, resulting in the oligomerization of this protease and its subsequent autocleavage and activation [45]–[48]. Recent studies have also suggested that alternative pathways may also be responsible for caspase-8 activation. Indeed, it has been observed that TGF-mediated caspase-8 activation in Burkitt cells does not involve death domain receptors [49], and FADD-independent caspase-8 activation by anticancer drugs has also been reported by Wesselborg et al [50].

**Figure 7:**
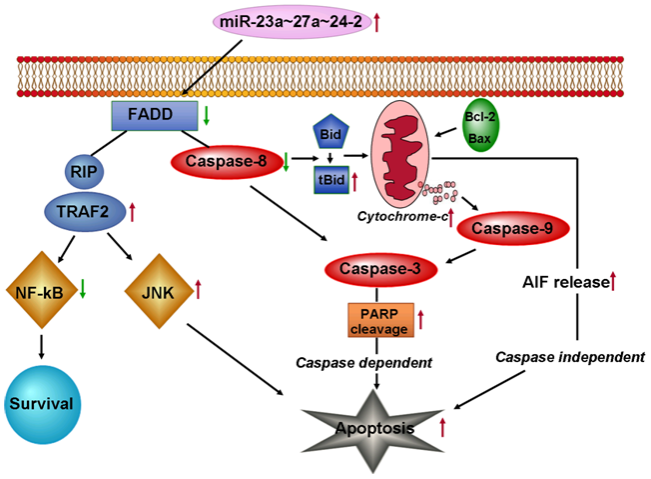
Hypothetical model for p(23a∼27a∼24-2) induced apoptosis. The green arrows indicate down regulation and red arrows indicate up regulation.

The data here suggests that there are at least two different pathways through which miR-23a∼27a∼24-2 cluster induces apoptosis in HEK293T cells: conventional caspase-dependent apoptotic cell death and caspase-independent cell death. Both occur via the mitochondrial membrane disruption pathway. Here, we propose a model (Fig. 7) for miR-23a∼27a∼24-2 cluster induced apoptosis in HEK293T cells. There is FADD-independent caspase-8 activation which in turn is followed by cleavage of Bid, the loss of mitochondrial membrane potential, induced reactive oxygen species (ROS) generation, release of apoptotic proteins from mitochondria, and subsequent caspase-dependent apoptosis. Our findings were confirmed by the upregulation of active form of caspase 3, down regulation of 116 kD PARP protein, Bcl-2, Bax and release of cytochrome-c from mitochondria to cytosol after over expression of miR-23a∼27a∼24-2 cluster in HEK293T cells. Rapid ROS generation might be causing the release of apoptosis inducing factor (AIF) from the mitochondria to the cytosol and nucleus, hence, leading to caspase 3-independent apoptosis by miR-23a∼27a∼24-2 cluster in HEK293T cells. There are several reports in the literature which support this fact [51], [52]. Further dissection of miR-23a∼27a∼24-2 induced apoptosis shows that there was increased TRAF2 expression after miR-23a∼27a∼24-2 cluster over expression. Literature suggests that trimerised TNFR1 can recruit proteins that engage various signal transduction pathways, some of which either abrogate or potentiate the apoptotic response [53] for example, the serine threonine kinase RIP and TRAF-2. Studies using RIP and TRAF-2 deficient cells indicate that RIP is most likely responsible for NF-κB activation, whereas, TRAF-2 preferentially activates c-Jun N- terminal kinase (JNK) [43]. Since in our study we observed increased TRAF-2 and decreased RIP levels we can hypothesize that in HEK293T cells TRAF-2 plays a major role in this cluster induced apoptosis. To prove this we did western blot analysis and observed that there was increased JNK activation as we observed increased p-JNK after miR-23a∼27a∼24-2 cluster over expression in HEK293T cells. We also did not observe any NF-κB activation and the NF-κB expression was found to be decreased after over expression of this cluster. In conclusion, we can say that over expression of miR-23a∼27a∼24-2 cluster induces caspase-dependent and independent apoptosis via JNK.

Our study extends the knowledge on FADD protein, a key death receptor adaptor molecule. In addition to death receptor ligand, FADD is also regulated at the transcriptional level by microRNA. Absence of FADD protein expression is a marker for tumor development and a prognostic factor for poor response to chemotherapy in humans. FADD deficient tumor cells resist death receptor-mediated apoptosis by chemotherapeutics drugs. Our data demonstrating that over expression of miR-23a∼27a∼24-2 sensitized HEK293T cells to TNF-α cytotoxicity could potentially be of value in cancer therapy.
